# Supplementary material for: Comparison of 46 Cytokines in Peripheral Blood Between Patients with Papillary Thyroid Cancer and Healthy Individuals with AI-Driven Analysis to Distinguish Between the Two Groups
Source: Diagnostics (Basel). 2025 Mar 20;15(6):791. doi: 10.3390/diagnostics15060791 (PMC11940922; doi:10.3390/diagnostics15060791)
Supplement: Supplementary file 1 [file diagnostics-15-00791-s001.zip › diagnostics-3478751-supplementary.pdf]

Supplementary Table S1. Intra-assay CV (%), inter-assay CV (%), and LLOQ (pg/mL) for each of the 46 cytokines.

Analyte	Intra-Assay (%CV)	Inter-Assay (%CV)	LLOQ (pg/mL)
CD40 Ligand	3.8	10.8	542.3
EGF	4.1	8.0	6.17
Eotaxin	4.8	7.4	19.09
FGF basic	2.0	7.6	11.82
Flt-3 Ligand	2.4	8.4	21.95
G-CSF	1.8	7.0	12.69
GM-CSF	3.4	11.2	17.94
Granzyme B	2.9	8.2	9.73
CXCL1/GRO α	1.9	5.6	18.24
CXCL2/GRO β	1.9	6.8	9.51
IFN-α2	3.6	10	4.43
IFN-β	2.4	7.1	3.07
IFN-γ	2.3	10.9	0.51
IL-1α	5.0	10.6	10.36
IL-1β	2.8	10.1	3.9
IL-1ra	3.6	12.4	10.32
IL-2	3.9	9.6	2.52
IL-3	3.6	9.9	16.65
IL-4	2.4	8.4	1.12
IL-5	1.8	8.3	6.69
IL-6	2.2	8.8	9.31
IL-7	2.8	9.6	3.88
IL-8	3.2	84	2.15
IL-9	6.2	10.3	364.75
IL-10	3.6	11.2	55.35
IL-12 p70	2.6	9.8	18.72
IL-13	2.5	8.3	29.41
IL-15	3.2	11.2	2.57
IL-17A	2.5	9.8	8.67
IL-17E	3.1	8.1	18.92
IL-33	2.9	9.7	13.99
CXCL10/IP-10	3.3	6.6	2.33
CCL2/MCP-1	1.8	6.3	4.12
CCL3/MIP-1α	2.9	5.7	4.58
CCL4/MIP-1β	1.7	7.9	74.49
CCL20/MIP-3α	3	9.4	2.33
CCL19/MIP-3β	2.5	8.6	4.46
PDGF-AA	2.7	7.1	5.49
PDGF-AB/BB	2.2	7.4	7.17
PD-L1/B7-H1	2.7	8.3	6.86
CCL5/RANTES	1.8	7.5	263.65
TGF-α	4.4	9.2	10.04
TNF-α	3.0	10.3	11.25
TNF-β	2.2	8.0	0.81

TRAIL	2.4	7.9	23.54
VEGF	2.9	10.1	6.67
Median	2.8	8.5	

Abbreviations: CD, cluster of differentiation; CV, coefficient of variance; CXCL, chemokine (C-X-C motif) ligand; EGF, epidermal growth factor; FGF, fibroblast growth factor; Flt, FMS-like tyrosine kinase; GM-CSF, granulocyte-macrophage colony-stimulating factor; IFN, interferon; IL, interleukin; IP, interferon-γ-induced protein; LLOQ, lower limit of quantification; MCP, monocyte chemoattractant protein; MIP, macrophage inflammatory protein; PDGF, Platelet-derived growth factor; PD-L1, programmed death ligand 1; ra, receptor antagonist; RANTES, regulated on activation, normal T cell expressed and secreted; TGF, transforming growth factor; TNF, tumor necrosis factor; TRAIL, tumor necrosis factor-related apoptosis-inducing ligand; VEGF, vascular endothelial growth factor

Supplementary Table S2. Assay working (measurement) range of each of the 46 cytokines.

**Analyte**	**Measurement Range (pg/mL)**
CD40 Ligand	542.3 ~ 395340.0
EGF	6.17 ~ 4500.0
Eotaxin	19.09 ~ 13920.0
FGF basic	11.82 ~ 8620.0
Flt-3 Ligand	21.95 ~ 16000.0
G-CSF	12.69 ~ 9250.0
GM-CSF	17.94 ~ 13080.0
Granzyme B	9.73 ~ 7090.0
CXCL1/GRO α	18.24 ~ 13300.0
CXCL2/GRO β	9.51 ~ 6930.0
IFN-α 2	4.43 ~ 3230.0
IFN-β	3.07 ~ 2240.0
IFN-γ	0.51 ~ 370.0
IL-1α	10.36 ~ 7550.0
IL-1β	3.9 ~ 2840.0
IL-1ra	10.32 ~ 7520.0
IL-2	2.52 ~ 1840.0
IL-3	16.65 ~ 12140.0
IL-4	1.12 ~ 820.0
IL-5	6.69 ~ 4880.0
IL-6	9.31 ~ 6790.0
IL-7	3.88 ~ 2830.0
IL-8	2.15 ~ 1570.0
IL-9	364.75 ~ 265900.0
IL-10	55.35 ~ 40350.0
IL-12 p70	18.72 ~ 13650.0
IL-13	29.41 ~ 21440.0
IL-15	2.57 ~ 1870.0
IL-17A	8.67 ~ 6320.0
IL-17E	18.92 ~ 13790.0
IL-33	13.99 ~ 10200.0
CXCL10/IP-10	2.33 ~ 1700.0
CCL2/MCP-1	4.12 ~ 3000.0
CCL3/MIP-1α	4.58 ~ 3340.0
CCL4/MIP-1β	74.49 ~ 54300.0
CCL20/MIP-3α	2.33 ~ 1700.0
CCL19/MIP-3β	4.46 ~ 3250.0
PDGF-AA	5.49 ~ 4000.0
PDGF-AB/BB	7.17 ~ 5230.0
PD-L1/B7-H1	6.86 ~ 5000.0
CCL5/RANTES	263.65 ~ 192200.0
TGF-α	10.04 ~ 7320.0
TNF-α	11.25 ~ 8200.0
TNF-β	0.81 ~ 590.0
TRAIL	23.54 ~ 17160.0
VEGF	6.67 ~ 4860.0

Abbreviations: CD, cluster of differentiation; CXCL, chemokine (C-X-C motif) ligand; EGF, epidermal growth factor; FGF, fibroblast growth factor; Flt, FMS-like tyrosine kinase; GM-CSF, granulocyte-macrophage colony-stimulating factor; IFN, interferon; IL, interleukin; IP, interferon-γ-induced protein; LLOQ, lower limit of quantification; MCP, monocyte chemoattractant protein; MIP, macrophage inflammatory protein; PDGF, Platelet-derived growth factor; PD-L1, programmed death ligand 1; ra, receptor antagonist; RANTES, regulated on activation, normal T cell expressed and secreted; TGF, transforming growth factor; TNF, tumor necrosis factor; TRAIL, tumor necrosis factor-related apoptosis-inducing ligand; VEGF, vascular endothelial growth factor

Supplementary Table S3. Coefficient of Variation in Nested Cross-Validation with 5 Inner Folds and 4-Fold/5-Fold Outer Folds.

Algorithms	ROC AUC	Accuracy	F1-score	Sensitivity (Recall)	Precision
**5-outer folds**					
XGBoost	2.42	8.88	8.78	5.46	4.95
SVM	8.89	6.13	14.73	17.31	7.93
NB	8.23	6.48	6.94	20.27	5.42
Log. Reg.	8.36	7.06	8.22	12.62	13.51
k-NN	9.49	10.06	12.65	23.58	11.75
**4-outer folds**					
XGBoost	3.06	3.59	3.55	5.71	5.79
SVM	5.64	7.35	8.49	13.19	5.78
NB	4.29	5.83	6.29	9.61	7.09
Log. Reg.	4.09	4.84	5.23	9.25	6.98
k-NN	11.40	9.05	10.50	18.84	8.69

Abbreviations: k-NN; k-nearest neighborhood algorithm, Log. Reg; Logistic Regression, NB; Naïve Bayes classification, SVM; Support Vector Machine, XGBoost; Extreme Gradient Boosting

Supplementary Table S4. Explained Variance by the Number of Data Dimensions.

Algorithms	ROC AUC	Accuracy	F1-Score	Sensitivity (Recall)	Precision	Specificity
k-NN	0.703	0.653	0.652	0.661	0.658	0.659
k-NN (reduced)	0.849	0.721	0.755	0.689	0.744	0.662
NB	0.919	0.841	0.840	0.847	0.840	0.835
NB (reduced)	0.934	0.873	0.861	0.876	0.850	0.867

Abbreviations: k-NN; k-nearest neighborhood algorithm, NB; Naïve Bayes Classifier

Supplementary Figure S1. Explained Variance by the Number of Data Dimensions.

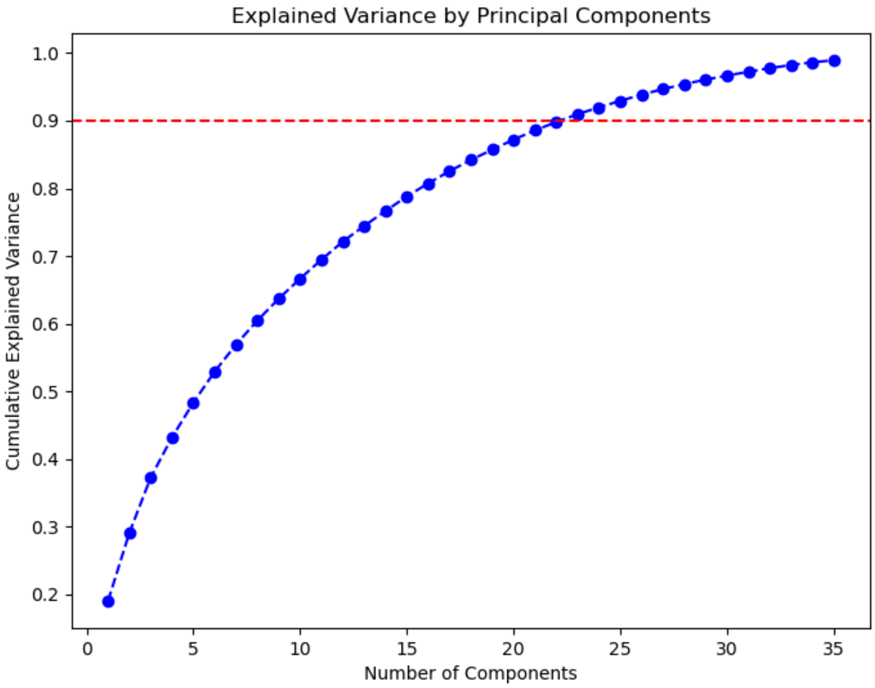

Supplementary Figure S2. Distribution of EGF, IL-10, CD40 Ligand, IL-1β in PTC and control groups.

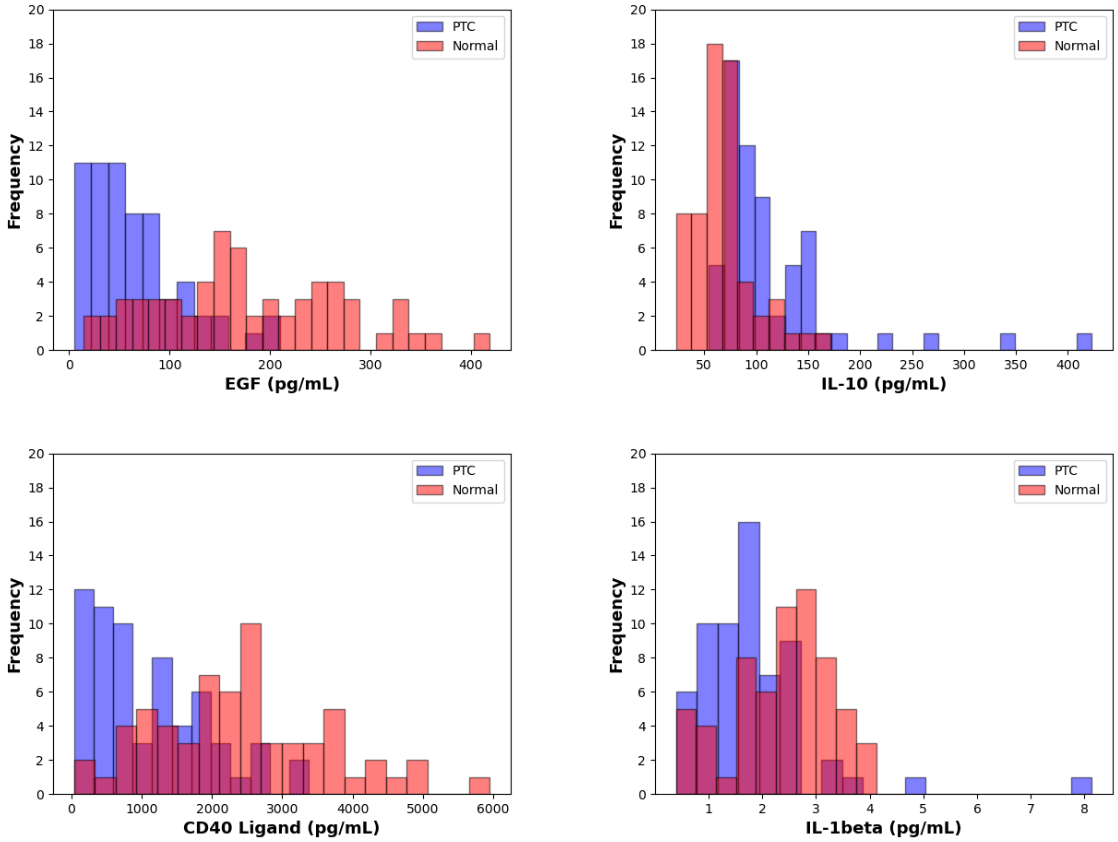

Abbreviations: EGF, epidermal growth factor; IL, interleukin;
